# Comparative genomic and functional analysis of *Arthrobacter* sp. UMCV2 reveals the presence of *luxR*-related genes inducible by the biocompound *N, N*-dimethylhexadecilamine

**DOI:** 10.3389/fmicb.2022.1040932

**Published:** 2022-10-31

**Authors:** Martha Patricia Chávez-Moctezuma, Ramiro Martínez-Cámara, Julie Hernández-Salmerón, Gabriel Moreno-Hagelsieb, Gustavo Santoyo, Eduardo Valencia-Cantero

**Affiliations:** ^1^Instituto de Investigaciones Químico Biológicas, Universidad Michoacana de San Nicolás de Hidalgo, Morelia, Michoacán, Mexico; ^2^Tecnológico Nacional de México, Morelia, Michoacán, Mexico; ^3^Department of Biology, Wilfrid Laurier University, Waterloo, ON, Canada

**Keywords:** actinobacteria, LuxR solos, domain approach, *airR* genes, *aiaR* genes, swarming motility

## Abstract

Quorum sensing (QS) is a bacterial cell-cell communication system with genetically regulated mechanisms dependent on cell density. Canonical QS systems in gram-negative bacteria possess an autoinducer synthase (LuxI family) and a transcriptional regulator (LuxR family) that respond to an autoinducer molecule. In Gram-positive bacteria, the LuxR transcriptional regulators *“solo”* (not associated with a LuxI homolog) may play key roles in intracellular communication. *Arthrobacter* sp. UMCV2 is an actinobacterium that promotes plant growth by emitting the volatile organic compound *N, N*-dimethylhexadecylamine (DMHDA). This compound induces iron deficiency, defense responses in plants, and swarming motility in *Arthrobacter* sp. UMCV2. In this study, the draft genome of this bacterium was assembled and compared with the genomes of type strains of the *Arthrobacter* genus, finding that it does not belong to any previously described species. Genome explorations also revealed the presence of 16 *luxR*-related genes, but no *luxI* homologs were discovered. Eleven of these sequences possess the LuxR characteristic DNA-binding domain with a helix-turn-helix motif and were designated as auto-inducer-related regulators (AirR). Four sequences possessed LuxR analogous domains and were designated as auto-inducer analogous regulators (AiaR). When swarming motility was induced with DMHDA, eight *airR* genes and two *aiaR* genes were upregulated. These results indicate that the expression of multiple *luxR*-related genes is induced in actinobacteria, such as *Arthrobacter* sp. UMCV2, by the action of the bacterial biocompound DMHDA when QS behavior is produced.

## Introduction

Quorum sensing (QS) is a bacterial cell-cell communication system based on the production, release, and detection of signal molecules called autoinducers (AI). QS regulates gene expression in response to changes in bacterial population density and produces coordinated behavior, based on environmental conditions (Fuqua et al., 1994; Waters and Bassler, 2005; Papenfort and Bassler, 2016). Cellular functions regulated by QS include bioluminescence, antibiotic synthesis, virulence factor production, biofilm induction, sporulation, and swarming motility (Polkade et al., 2016; Bramhachari et al., 2018).

The *lux* operon, responsible for bioluminescence in the model organism *Vibrio fischeri* (*Aliivibrio fischeri*), is composed of six structural genes (*luxA-E* and *luxG*) and the regulatory genes *luxI* and *luxR* that encode homonym proteins (Fuqua et al., 1996; Miller and Bassler, 2001). LuxI is an acyl-synthase of approximately 190 amino acids that uses the acyl carrier protein as a substrate to add the *N*-acyl group to *L*-homoserine lactone derived from *S*-adenosylmethionine and produce acyl-homoserine lactones (AHLs)., the main autoinducers in Gram-negative bacteria. In other bacteria, LuxI homologs add different *N*-acyl groups to *l*-homoserine lactones to produce different AHLs (Fuqua et al., 1994; Zavilgelsky and Manukhov, 2001). LuxR is a transcriptional regulator of approximately 250 amino acids with characteristic modular architecture consisting of an autoinducer binding domain in the N-terminal region (Shadel et al., 1990; Slock et al., 1990) and a DNA-binding domain with a helix-turn-helix (HTH) motif in the C-terminal region (Choi and Greenberg, 1991; Fuqua et al., 1994).

Quorum sensing systems were identified in both Gram-negative and Gram-positive bacteria (Hawver et al., 2016). In Gram-negative bacteria, the more studied QS systems are regulated by members of the AHL family (Swift et al., 1994) which act as diffusible signal molecules, whose synthesis is performed by members of the LuxI family. Above a threshold concentration, these molecules bind to members of the transcriptional regulator LuxR family (Fuqua et al., 1996). The stable LuxR-AHL complex subsequently binds to the *lux box*, a specific regulatory sequence in the promoter region, to activate or suppress the transcription of its target genes (Fuqua et al., 2001).

The analysis of different proteobacterial genomes has revealed the wide presence of homologs of the transcriptional regulator luxR without the presence of their corresponding homolog LuxI, thus referred to as luxR orphans or “*solos*” (Fuqua, 2006; Case et al., 2008; Patankar and González, 2009). These transcriptional regulators possess the same structural organization as canonical *LuxR* (Bez et al., 2021). *Stenotrophomonas maltophilia* encodes the transcriptional regulator SmoR, a non-LuxI-associated LuxR homolog that binds to oxo-C8-homoserine lactone produced by *Pseudomonas aeruginosa*. SmoR regulates its operon transcription and promotes swarming motility in *S. matophilia* (Martínez et al., 2015). LuxR solo proteins are involved in inter-kingdom signaling, responding to the signals produced by eukaryotes (Venturi and Fuqua, 2013; Kan et al., 2017). A subfamily of luxR is present exclusively in plant-associated bacterial (PAB) responses to plant low-molecular weight compounds (González et al., 2013; González and Venturi, 2013; Venturi and Fuqua, 2013). Structurally, PAB-LuxR is very similar to canonical LuxR but differs in one or two of the aromatic or hydrophobic amino acids normally conserved in the auto-inducer binding domain of canonical LuxR proteins (González and Venturi, 2013). PAB-LuxR proteins are present in phytopathogenic bacteria as XccR of *Xanthomonas campestris* (Zhang et al., 2007), or OryR of *Xanthomonas oryzae* (Ferluga and Venturi, 2009) and in plant beneficial bacteria, such as *Pseudomonas* sp. GM79 (Coutinho et al., 2018) and PsrR of *Kosakonia* sp. KO348 (Mosquito et al., 2020). Other LuxR proteins respond to endogenous molecules other than AHLs and are therefore not associated with LuxI synthases. PluR, present in *Photorhabdus luminescens*, responds to α-pyrones produced by the ketosynthase PpyS, whereas PauR of the human pathogen *Photorhabdus asymbiotica* senses dialkylresorcinols and cyclohexanediones produced by the bacteria (Brachmann et al., 2013; Brameyer et al., 2014, 2015).

Other molecules, such as volatile organic compounds (VOCs) produced by bacteria, act as signaling molecules regulating processes that are frequently regulated by QS (Schulz-Bohm et al., 2017; Xie et al., 2018). Resistance against antibiotics, virulence, and motility are examples of behaviors regulated by bacterial VOCs. The mechanisms involved in the communication mediated by VOCs and the regulation of these behaviors are primarily unknown (Kim et al., 2013; Enkataraman et al., 2014; Mansurova et al., 2018). The participation of luxR transcriptional regulators is not discarded.

In gram-positive bacteria, QS systems are usually regulated by auto-inducing peptides (Monnet and Gardan, 2015; Hawver et al., 2016; Aframian and Eldar, 2020), released by specific transporters into the extracellular media, where they are detected by a transmembrane receptor. Autoinducer detection triggers a signaling pathway through the successive phosphorylation/dephosphorylation of a sensing transmembrane histidine kinase and a transcriptional regulator that interacts with DNA and controls the QS response (Lyon and Novick, 2004; Dufour and Lévesque, 2013; Monnet et al., 2014). Although LuxR-LuxI systems are marginally present in Gram-positive bacteria, *luxR* solo genes are frequently found in that group of bacteria (Rajput and Kumar, 2017).

*Arthrobacter* sp. UMCV2 is an actinobacterium (Gram-positive) isolated from the *Zea mays* rhizosphere (Valencia-Cantero et al., 2007). This *Arthrobacter* strain promotes plant growth through the emission of VOCs, predominantly *N, N*-dimethylhexadecylamine (dimethylhexadecylamine, DMHDA) (Velázquez-Becerra et al., 2011). DMHDA emitted by *Arthrobacter* sp. UMCV2 accumulates in the extracellular medium and modulates the growth and swarming motility of *Arthrobacter* sp. UMCV2 and other bacteria (Velázquez-Becerra et al., 2013; Martínez-Cámara et al., 2020). In this study, we analyzed the *Arthrobacter* sp. UMCV2 genome, identified and classified the *Arthrobacter* sp. UMCV2 *luxR*-related genes, and demonstrated the upregulation of *luxR*-related genes by DMHDA when motility-associated genes are induced.

## Materials and methods

### Biological material

The strain used in this study was *Arthrobacter* sp. UMCV2 (Valencia-Cantero et al., 2007), deposited at the Microorganism Collection of the National Center of Genetic Resources (Boulevard de la Biodiversidad 400, Rancho las Cruces, 47600 Tepatitlán de Morelos, Jalisco, México) having accession number CM-CNRG-691. The bacterial strain was routinely cultured on nutrient agar (NA) or nutrient broth (NB) at 22^°^C.

### Chemicals

The chemical compound *N, N*-dimethylhexadecylamine (DMHDA) was purchased from Sigma Aldrich (St Louis MO USA, catalog 40460) and dissolved in ethanol. Equal volumes of solvent were used in all the treatments.

### Genomic DNA extraction

From a single colony culture, *Arthrobacter* sp. UMCV2 was inoculated into 50 ml of NB at 22^°^C with agitation at 180 rpm for 2 days. Genomic DNA was extracted from 20 ml of liquid culture using the Wizard Genomic DNA Purification Kit (Promega, Madison, WI, USA, catalog A1120), according to the manufacturer’s instructions. The quality and quantity of genomic DNA were assessed by agarose gel electrophoresis using a NanoDrop 1000 (Thermo Scientific, Rockford, IL, USA).

### Genome sequencing, assembly, annotation, and phylogenetic analysis

The genome of *Arthrobacter* sp. UMCV2 was sequenced at the genomic sequencing facilities of LANGEBIO, Cinvestav-IPN (Irapuato-México), using an Illumina MiSeq platform generating three paired-end libraries, with coverage of 57x, the original data were assembled into 461 contigs or scaffolds using Newbler v2.9 software. To order the contigs, *A. chlorophenolicus* A6 was used as the reference genome. A summary of the project information is presented in Supplementary Table 1. The genome sequence was submitted to the RAST web service (MG-RAST 4.0.3)^1^ for automated annotation (Aziz et al., 2008). The sequence was deposited in the GenBank database of the National Center for Technology Information (NCBI)^2^ with accession number (CP024915.1).

A genomic-based phylogenetic tree, a 16S-based phylogenetic tree and digital DNA–DNA hybridization (dDDH) were performed with the assembled genome using the Type Strain Genome Server (TYGS) pipeline (Meier-Kolthoff and Göker, 2019; Meier-Kolthoff et al., 2022).^3^ Trees were inferred using FastME 2.1.6.1 from Genome BLAST Distance Phylogeny (GBDP), distance formula d5 (Farris, 1972; Meier-Kolthoff et al., 2013; Lefort et al., 2015), comparing the genomes of *Arthrobacter* sp. UMCV2, with type strains deposited in the DSMZ database.

### *In silico* analysis of *Arthrobacter* sp. UMCV2 *luxR*-related genes

In addition to NCBI gene notation, a complementary search for *luxR* and corresponding *LuxI* homologous genes was performed with RAST MG-RAST 4.0.3 (see above) and PATRIC 3.6.12 (Brettin et al., 2015).^4^ Putative *luxR* homologs were analyzed using InterPro v62.0 (Blum et al., 2021).^5^

Phylogenetic and molecular evolutionary analyses were conducted using MEGA version X (Kumar et al., 2018) using the maximum likelihood method and the Jones-Taylor-Thornton (JTT) matrix-based model (Jones et al., 1992). The bootstrap consensus tree inferred from 1,000 replicates was used to represent the evolutionary history of the taxa analyzed (Felsenstein, 1985). Initial trees for the heuristic search were obtained automatically by applying the Neighbor-Join and BioNJ algorithms to a matrix of pairwise distances estimated using the JTT model followed by selecting the topology with a superior log-likelihood value. The analysis involved 44 amino acid sequences. There were 1,098 positions in the final dataset.

### Bacterial motility assay

The effect of DMHDA on bacterial motility was tested on NA plates with DMHDA at concentrations of 0, 0.1, 0.2, 3.0, 4.0, 5.0, and 6.0 μM. Subsequently, 5 μl of bacterial suspension (OD_595_ = 1) was added to each plate center. The swarming motility was assessed following the protocols described by Martínez-Cámara et al. (2020) with a few modifications, such as petri dishes prepared with NB plus 0.5% agar and incubated for 120 h at 23^°^C. The motility diameter was measured using a digital Vernier caliper (Mitutoyo Corporation, Tokyo Japan, catalog 500-196-30) and the results were reported in centimeters of bacterial extension on the media. Three independent assays, each with four replicates, were performed.

### Gene expression analysis

RNA extraction was performed for three biological replicates of *Arthrobacter* sp. UMCV2 from swarming motility, as previously described (Martínez-Cámara et al., 2020). RNA was extracted using TRI reagent (Sigma Aldrich, St Louis, MO, USA, catalog T9424) and treated with DNase I to remove the remaining genomic DNA. The integrity of the RNA was assessed by visualization on 1.2% agarose gel. The final concentration was estimated using NanoDrop (Thermo Scientific, Rockford, IL, USA). cDNA synthesis was performed using 500 ng RNA template and SuperScript First-Strand Synthesis System (Life Technologies/Gibco-BRL CA, USA). Reverse transcription quantitative real-time PCRs (RT-qPCR) were performed on an ABI StepOneTM System thermocycler (Applied Biosystems; Foster City, CA, USA). Oligonucleotides were designed (using the NCBI for Biotechnology Information primer design tool) to amplify *airR1*-*12* and *aiaR1-4* (Supplementary Table 2). Oligonucleotides to amplify *fliC*, *flgL*, *fliM, motA*, and *recA* have been previously reported (Martínez-Cámara et al., 2020), *recA* was used as the normalizer gene. RT-qPCR analysis was performed using the SYBR-Green kit (Applied Biosystems, Foster City, CA, USA, catalog 4385612) and the following protocol: 5 μl SYBR Green, 1 μl direct and inverse oligo, 2 μl cDNA, and 1 μl water. Samples were run using the following protocol: 95^°^C for 5 min, 40 cycles of 95^°^C for 15 s, and 60^°^C for 1 min. To prepare the melting curve, samples were run at 95^°^C for 15 s and 60^°^C for 1 min, and the temperature was subsequently raised to 95^°^C at a rate of 0.3^°^C/s. Gene expression was evaluated using the comparative ^ΔΔ^ Ct method according to Livak and Schmittgen (2001).

### Statistical analysis

The results were analyzed using Student’s *t*-test or analysis of variance and Duncan’s multiple range test for multiple (*p* ≤ 0.05).

## Results

### Genome properties

**FIGURE 1:**
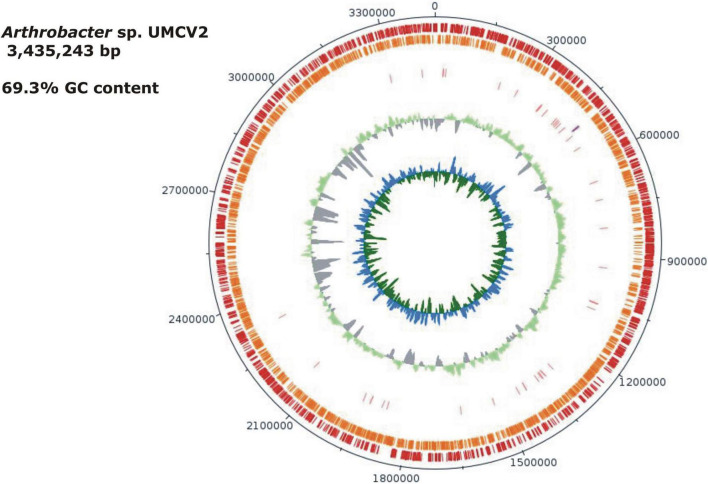
Graphical map of the *Arthrobacter* sp. UMCV2. From outside to the center: genes on the forward strand (red), genes on the reverse strand (orange), RNA genes (pink tRNAs, purple rRNAs), the guanine-cytosine (GC) ratio is in light green and gray. The inner circle shows the GC skew in blue and green. Gaps between individual contigs are not presented. The order of the contigs was assumed to correspond to a reference genome. Contigs not matching the reference genome were ordered from the largest to the smallest. Ordered contigs were joined with 50 bp of “N.” This figure was drawn with the DNA plotter software.

**FIGURE 2:**
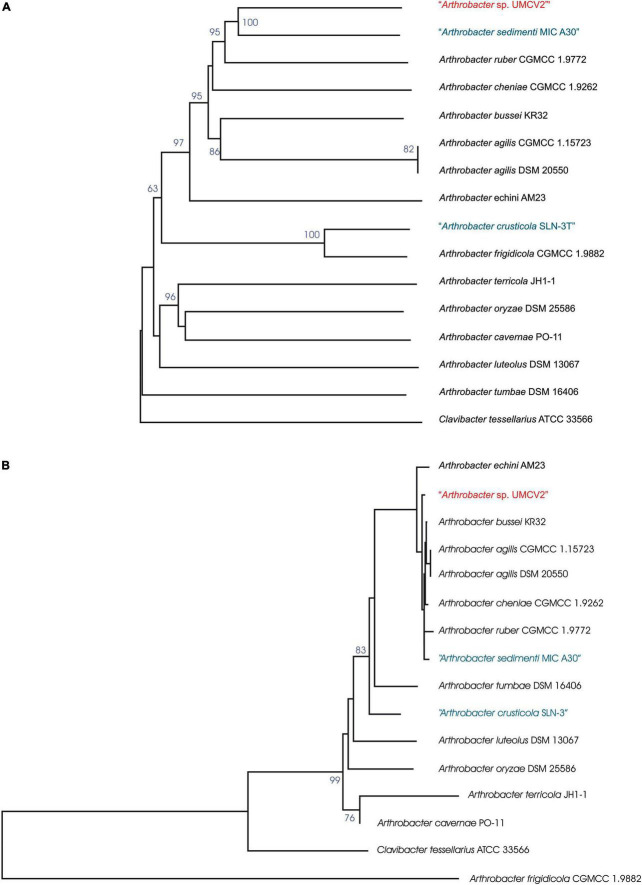
*Arthrobacter* sp. UMCV2 phylogenetic tree based on the genomic **(A)** and **(B)** 16S rRNA gene sequences. The trees were inferred from genome BLAST distance phylogeny (GBDP) distances. The branch lengths were scaled in terms of GBDP distance formula d5. The numbers above branches are GBDP pseudo-bootstrap support values >60% from 100 replications, with average branch support of 77.4% for genomic tree and 57.2% for 16S rRNA gene sequences tree. The trees were rooted at the midpoint. Species in blue were mentioned as not validly published by the DSMZ server https://lpsn.dsmz.de/ at the time of writing.

The *Arthrobacter* sp. UMCV2 assembled genome sequence consists of a single circular chromosome of 3,435,243 bp with a 69.3% of GC content (Figure 1). Automatic gene functional annotation predicted 3,114 genes, consisting of 3,067 protein-coding and 47 RNA genes (Table 1). The gene distribution had representatives of 23 “Clusters of Orthologous Groups” (COGs) functional categories (Supplementary Table 3). The genome size of *Arthrobacter* sp. UMCV2 is similar to the genome size of closed-related type strains of *Arthrobacter* species deposited in the DSMZ database (Supplementary Table 4) that range between 3.17 Gb of *A. echini* and 4.42 Gb of *A. sedimenti*. Genomic-based and 16S-based phylogenetic trees were generated using the complete genome sequences. 16S genes of the closed-related bacterial type strain deposited in the DSMZ database were detected using the pipeline of the SYGS server (Meier-Kolthoff and Göker, 2019). Both phylogenetic trees presented a clear affiliation of the UMCV2 strain with the *Arthrobacter* genus. However, further genomic (Figure 2A) or 16S (Figure 2B) phylogenetic trees did not conclusively supported a taxonomic position at the species level. The 16S-based phylogenetic tree showed clustering of the *Arthrobacter* sp. UMCV2 strain with the bacterial species *A. echini*, *A. bussei*, *A. agilis*, *A. cheniae*, *A. ruber*, and *A. sedimenti*. The genomic-based phylogenetic tree retains the same type of clustering and clearly shows a divergence of the UMCV2 branch from the other *Arthrobacter* species.

**TABLE 1:** *Arthrobacter* sp. UMCV2 genome statistics.

Attribute	Value	% of total
Genome size (bp)	3,435,243	100
DNA coding region (bp)	2,735,154	79.62
DNA G + C content (bp)	1,902,572	69.3
DNA scaffolds	461	100
Total genes	3,114	100
Protein coding genes	3,067	98.49
RNA genes	47	1.51
Genes assigned to COGs	1,884	60.50
Genes with Pfam domains	1,930	61.95

Relatednesses between the genomes of *Arthrobacter* sp. UMCV2 and closed-related type strains of *Arthrobacter* species were calculated as dDDH percentages. Concordantly with the phylogenetic tree topologies, the highest dDDH values of *Arthrobacter* sp. UMCV2 were produced with cluster members of type strains such as *A. bussei*, *A. agilis*, *A. cheniae*, *A. ruber*, and *A. sedimenti*, ranging from 40.3% (with *A. ruber*) to 36.2% (with *A. bussei*). Similar dDDH values were produced among type strains of the same cluster ranging from 47% (*A. agilis* to *A. bussei*) to 31.4% (*A. agilis* to *A. sedimenti*) (Table 2). These results strongly suggest that *Arthrobacter* sp. UMCV2 belongs to a species not yet described within the genus *Arthrobacter*.

**TABLE 2:** Genomic relatedness matrix of *Arthrobacter* sp. UMCV2 and closed-related type strains calculated as digital DNA–DNA hybridization (dDDH) percentages^a^.

Bacterial strain^b^	*Arthrobacter* sp. UMCV2	*Arthrobacter sedimenti* MIC A30 ^T^	*Arthrobacter ruber* CGMCC 1.9772^T^	*Arthrobacter cheniae* CGMCC 1.9262^T^	*Arthrobacter bussei* KR32^T^	*Arthrobacter agilis* CGMCC 1.15723	*Arthrobacter agilis* DSM20550^T^	*Arthrobacter echini* AM23^T^	*Arthrobacter frigidicola* CGMCC 1.9882^T^	*Arthrobacter crusticola* SLN-3T^T^	*Arthrobacter cavernae* PO-11^T^	*Arthrobacter oryzae* DSM 25586^T^
*Arthrobacter* sp. UMCV2	−											
*Arthrobacter sedimenti* MIC A30^T^	36.7	−										
*Arthrobacter ruber* CGMCC 1.9772^T^	40.3	35.8	−									
*Arthrobacter cheniae* CGMCC 1.9262^T^	34.5	34.9	38.6	−								
*Arthrobacter bussei* KR32^T^	36.2	32.4	38.1	37.6	−							
*Arthrobacter agilis* CGMCC 1.15723	37.3	31.4	38.7	36.5	46.9	−						
*Arthrobacter agilis* DSM20550^T^	37.3	31.4	38.6	36.4	47.0	100	−					
*Arthrobacter echini* AM23^T^	23.7	23.3	26.9	22.0	24.7	25.2	25.2	−				
*Arthrobacter frigidicola* CGMCC 1.9882^T^	18.4	18.6	20.2	18.4	18.7	19.0	19.0	17.0	−			
*Arthrobacter crusticola* SLN-3T^T^	19.1	19.2	20.6	18.7	19.2	19.2	19.2	17.4	70.8	−		
*Arthrobacter cavernae* PO-11^T^	14.4	14.6	14.7	14	14.3	14.1	14.5	13.9	14.5	14.5	−	
*Arthrobacter oryzae* DSM 25586^T^	14.3	14.6	14.6	13.9	14.4	14.3	14.3	13.8	14.5	14.4	20.8	−
*Arthrobacter luteolus* DSM 13067^T^	14.2	14.2	14.4	13.7	14.3	14.2	14.2	14.1	14.5	14.8	14.5	14.3

^a^dDDH vales were calculated according to the formula one of the type strain genome server (TYGs) pipeline as described by Meier-Kolthoff and Göker (2019).

^b^Type bacterial strains are marked with^T^.

### Identification of characteristic LuxR domains

**FIGURE 3:**
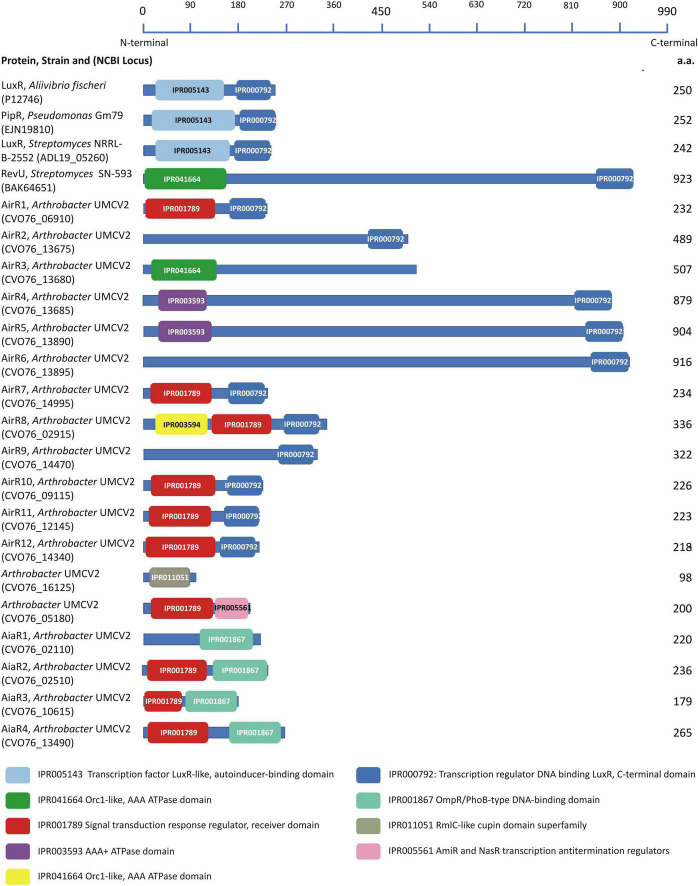
Conserved domains found in the model LuxR proteins and LuxR-related sequences from *Arthrobacter* sp. UMCV2. LuxR-related sequences were localized to the genome of *Arthrobacter* sp. UMCV2 employs the informatic tools MG-RAST 4.0.3 and PATRIC 3.6.12. Domains were localized in the protein sequences employing the informatics tool InterPro v62.0. Proteins are represented with a blue line and their lengths are compared with the upper scale. Conserved domains are localized in the proteins with colored boxes. Protein names, the strain of origin, and national center for technology information (NCBI) locus are indicated in the left column.

Analysis of the *Arthrobacter* sp. UMCV2 genome was performed with the MG-RAST and PATRIC servers and 18 putative *luxR* homolog sequences, but no *LuxI* putative homologs were identified. Protein sequences corresponding to the putative *luxR* homologs were examined to identify LuxR-characteristic domains using the InterPro server. *Aliivibrio fischeri* LuxR (Fuqua et al., 1994) and *Pseudomonas* sp. GM79 PipR (Coutinho et al., 2018) were included in the analysis as references for canonical experimentally characterized LuxR proteins, *Streptomyces purpurogeneiscleroticus* NRRL B-2952 LuxR (Rajput and Kumar, 2017) as a canonical *in silico* characterized LuxR protein from actinobacteria, and *Streptomyces* SN-593 RevU (Panthee et al., 2020) as an experimentally characterized LuxR-related protein from actinobacteria. All four reference proteins presented a characteristic DNA-binding domain at the C-terminal end (IPR000792). The canonical LuxR proteins displayed an autoinducer binding domain (IPR005143) at the N-terminal end, whereas RevU (LAL-LuxR protein) *Streptomyces* SN-593 exhibited an alternative ATPase domain (IPR041664) (Figure 3). Interestingly, the length of the RevU protein was significantly longer (923 a.a.) than the canonical LuxR (250 a.a.).

Further, we analyzed 18 *Arthrobacter* sp. UMCV2 putative LuxR proteins for conserved domains (Figure 3). Eleven sequences showed the characteristic DNA-binding domain IPR000792 at the C-terminal end (designated as *airR* sequences), four showed the IPR001867 domain described as a DNA-binding domain type OmpR/PhoB (designated as aiaR sequences), one sequence contained the IPR005561 domain described as transcription antitermination regulator, and two sequences did not show conserved C-terminal domains (Figure 2). At the N-terminal end, 10 sequences matched the IPR001789 domain, described as a signal transduction response regulator receiver domain, three sequences matched the ATPase domains (IPR041664 or IPR003593), and three sequences did not show conserved domains at the N-terminal end. The putative LuxR homologs in *Arthrobacter* sp. UMCV2 did not contain the canonical LuxR autoinducer-binding domain IPR005143. Only one sequence earlier recognized as a putative LuxR homolog sequence showed the unrelated domain IPR011051 (RmlC-like cupin domain superfamily). Similar to RevU from *Streptomyces* SN-593, five sequences were significantly longer (489–926 a.a.) than canonical LuxR sequences. Sequences CVO76_16125 and CVO76_05180 with the domains IPR011051 and IPR005561, respectively, were discarded as LuxR-related since their domains are discordant with LuxR proteins (Figure 3).

### Evolutionary relationships of LuxR-related sequences

**FIGURE 4:**
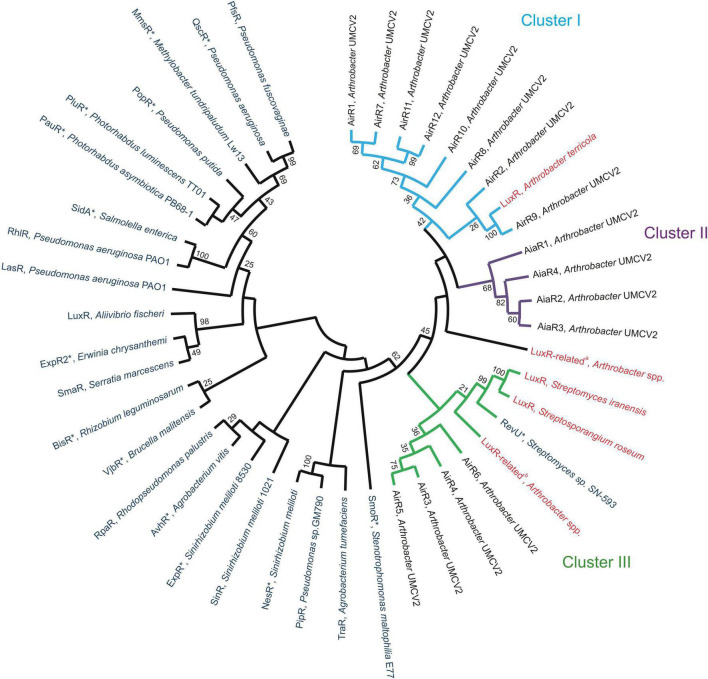
Evolutionary relationships among *Arthrobacter* LuxR-related sequences. The evolutionary relationship was inferred using the Maximum Likelihood method and jones-taylor-thornton (JTT) matrix-based model. The bootstrap consensus tree was inferred from 1,000 replicates. Blue sequences have been experimentally characterized, red denotes sequences informatically characterized and black is *Arthrobacter* sp. UMCV2 sequences established in this work. Cluster I (light blue, sequences with domains IPR001789, and IPR000792 or IPR000792 alone), Cluster II (purple, sequences with domains IPR001789, and IPR001867), and Cluster III (green, larger proteins) are emphasized. Accession numbers of employed sequences are provided in Supplementary Table 5. LuxR *solos* are marked with *.

LuxR-related sequences were used to construct a phylogenetic tree to observe their association with previously experimentally or *in silico* characterized LuxR-related sequences (Figure 4 and Supplementary Table 6). All Actinobacteria LuxR-related proteins clustered together with the 16 putative LuxR homologs from *Arthrobacter* sp. UMCV2 (AirR and AiaR sequences), three LuxR or LuxR-related sequences from *Streptomyces* and *Streptosporangium*, and three luxR or luxR-related sequences from *A. terricola* or *Arthrobacter* species. Whereas all the other analyzed luxR-related sequences from Proteobacteria clustered together with LuxR of *A. fischeri* (Figure 4). Among the LuxR-related sequences of *Arthrobacter* sp. UMCV2, three principal clusters were observed. Cluster I grouped proteins of approximately 218–439 a.a. with the concurrence of the domains IPR001789 and IPR000792 (six proteins) or the IPR000792 domain alone (two proteins). LuxR from *Arthrobacter terricola* (previously characterized *in silico*) belonged to this cluster (Figure 4). Cluster II grouped proteins with the domain IPR001867 (the four AiaR proteins) and Cluster III grouped larger proteins (507–916 a.a.) with the LuxR-related proteins of *Streptomyces, Streptosporangium*, and *Arthrobacter* multispecies (Figures 3, 4). We included sequences of LuxR associated with LuxI and LuxR solos, although no preferential clustering between *luxR* solo genes was observed. These results indicate that the 16 analyzed sequences from *Arthrobacter* sp. UMCV2 belongs to the Actinobacteria group of LuxR-related sequences separate from the canonical LuxR from Proteobacteria, suggesting early divergence between these transcriptional regulators in both phyla.

### *N, N*,-dimethylhexadecylamine modulates *Arthrobacter* sp. UMCV2 swarming motility

**FIGURE 5:**
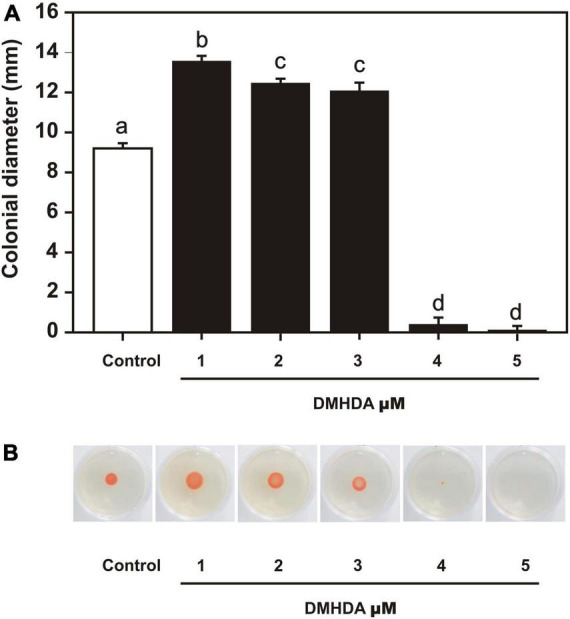
The effect of dimethylhexadecylamine (DMHDA) on *Arthrobacter* sp. UMCV2 swarming motility. Nutrient agar plates were prepared with various concentrations of DMHDA and inoculated with 5 μl bacterial suspension. After 72 h of culturing, colony diameters were measured. Bars, and error bars on panel **(A)**, represent the mean ± standard error values, respectively, for four biological replicates. Letters indicate means that differ significantly, following Duncan’s multiple range test (*p* < 0.05). Panel **(B)** shows representative images of the treatments.

*Arthrobacter* sp. UMCV2 was placed in NB plates supplemented with DMHDA 1.0–6.0 μM to observe the bacterial swarming motility. After 72 h, *Arthrobacter* sp. UMCV2 showed an enhancement of 66, 61, and 59% in swarming motility on plates at 1.0, 2.0, and 3.0 μM DMHDA, respectively, compared with controls although, a drastic motility decrease was observed on plates with 4.0 and 5.0 μM DMHDA (Figure 5). These results show that DMHDA modulates swarming motility, a behavior usually associated with quorum sensing signaling that involves the bacterial flagellar apparatus.

### *N, N*,-dimethylhexadecylamine modulates the expression of auto-inducer related regulators and auto-inducer analog regulator genes

**FIGURE 6:**
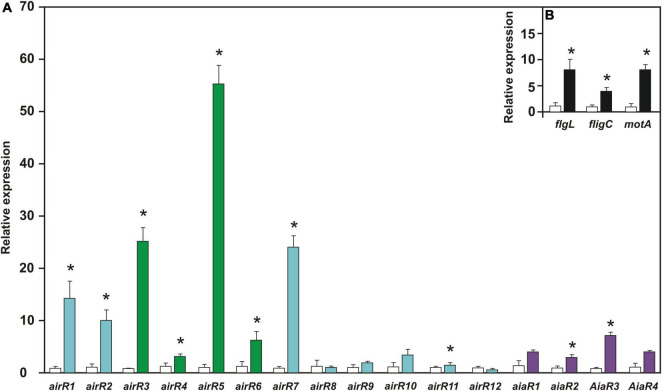
The relative expression of auto-inducer-related regulators (*airR*), auto-inducer analog regulator (*aiaR*), and motility-involved genes in *Arthrobacter* sp. UMCV2 induced with DMHDA. *Arthrobacter* sp. UMCV2 was induced with dimethylhexadecylamine (DMHDA). After 72 h, relative expression of *airR*, *aiaR* genes panel **(A)**, and motility-involved genes panel **(B)** were determined. Values represent mean ± standard errors of relative expression of three biological replicates in reference to control (with bars). Asterisks indicate significant differences between treatments calculated using Student’s *t*-test (*p* < 0.05). Genes belonging to Cluster I, II, and III of Figure 4 are colored in light blue, purple, and green, respectively.

The expression of *airR* and *aiaR* genes, together with the flagellar genes *flgL*, *fliC*, and *motA*, were quantified in bacterial growth with 1.0 μM DMHDA and compared with the respective controls (0.0 μM). Eigth of the *airR* and two of the *aiaR* genes exhibited statistically significant induction. The overexpression of *airR5* was 55-fold, followed by *airR3* and *airR7*, over expressing by 25-fold. The overexpression of *airR1*, *airR2*, *airR6*, and *aiaR3* ranged between 14- and 6-fold. The overexpression of *airR11*, *aiaR2*, and *aiaR3* ranged between 4- and 1.6-fold (Figure 6A). With the increase in swarming motility, the expression of flagellar genes *flgL*, *fliC*, and *motA* also increased, showing an overexpression ranging from 8- to 4-fold (Figure 6B). These results showed that DMHDA modulates the expression of different *airR* and *aiaR* genes at different magnitudes, simultaneous with flagellar genes.

## Discussion

*Arthrobacter* sp. UMCV2 is a plant growth-promoting rhizobacterium that was isolated while screening for iron-reducing bacteria, capable of supplying ferrous iron to plants in alkaline soils where this metal is limited to plant growth and development (Valencia-Cantero et al., 2007). This beneficial bacterium also promotes plant growth through the emission of the volatile organic compound DMHDA (Velázquez-Becerra et al., 2011). DMHDA acts as an inter-kingdom signaling molecule interacting with the plant cytokinin signaling pathway involving the AHK2 receptor (Vázquez-Chimalhua et al., 2021a) and producing in this way, a cross-talk with the jasmonic-acid pathway (Vázquez-Chimalhua et al., 2019). Plant organogenesis and growth are modulated by DMHDA through modifying the balance between stem cell niche and Jasmonic acid-dependent gene expression (Vázquez-Chimalhua et al., 2021b). DMHDA also induces expression of genes involved in plant systemic defense mechanisms and Fe-acquisition strategies, which are suggested to enhance photosynthesis and biomass production (Orozco-Mosqueda et al., 2013; Castulo-Rubio et al., 2015; Montejano-Ramírez et al., 2020). Previous results showed that *Arthrobacter* sp. UMCV2 adopts an endophytic lifecycle and colonizes plant tissues, providing benefits to host plants (Montejano-Ramírez et al., 2018; Hernández-Soberano et al., 2020).

### *Arthrobacter* sp. UMCV2 genome and taxonomy

*Arthrobacter* sp. UMCV2 was initially identified as *Arthrobacter agilis* since its 16S rRNA sequences showed a percent identity above 99.5% (Valencia-Cantero et al., 2007). However, the 16S rRNA sequences of *A. echini* (Lee et al., 2016), *A. ruber* (Liu et al., 2018), *A. bussei* (Flegler et al., 2020), *A. sedimenti* (Lin et al., 2020), and *A. cheniae* (Yang et al., 2021) also share a percent identity above 99.5% with the 16S rRNA gene of *Arthrobacter* sp. UMCV2. In this study, the TYGS pipeline was employed to produce phylogenetic trees of the type strains of the species most closely related to *Arthrobacter* sp. UMCV2 on 16S rRNA and genomic terms (Figure 2), to determine the specific taxonomic position of *Arthrobacter* sp. UMCV2. Although our results place UMCV2 in the *Arthrobacter* genus, *Arthrobacter* sp. UMCV2 is not included in the *A. agilis* branch or in branches together with other type strains. Then, we used a dDDH approach to calculate the relatedness between the genomes of *Arthrobacter* sp. UMCV2 and type strains of closely related species. dDDH is a recognized standard procedure for genome sequence-based species delimitation (Meier-Kolthoff et al., 2013). Thus, we found that the higher dDDH values were shared between *Arthrobacter* sp. UMCV2 and a cluster of type strains including *A. bussei*, *A. agilis*, *A. cheniae*, *A. ruber*, and *A. sedimenti*, ranging from 40.3 to 36.2%, which are clearly below the 70%, which is generally accepted as the species boundary (Chun et al., 2018); concordantly, dDDH values detected among type strains were similarly low (Table 2). Therefore, we conclude that *Arthrobacter* sp. UMCV2 might be a new species that can eventually be described.

The *Arthrobacter* sp. UMCV2 genome has a typical size for the *Arthrobacter* genus. The genes involved in survival and colonization, such as *SOD*, showed aptitudes for colonization of roots (Kim et al., 2000; Alquéres et al., 2013), in addition to the operon *ars*, which confers resistance to arsenic and arsenate, a toxic metalloid (Wang et al., 2009). The presence of *fhuDCB* genes can be important in siderophore piracy (Koster, 1991; Galet et al., 2015), which is important for competition in soil.

### *Arthrobacter* sp. UMCV2 possesses *luxR*-related genes

The presence of *luxR* annotated genes was particularly interesting since *luxR* genes are involved in the QS process. Previously, we found that DMHDA induces swarming motility in *Arthrobacter* sp. UMCV2, frequently recognized as a behavior regulated by QS (Martínez-Cámara et al., 2020). The canonical *luxI*-*luxR* system is typically the central component of QS in Gram-negative bacteria (Whiteley et al., 2017), but is exceptionally present in Gram-positive bacteria (Rajput and Kumar, 2017). Additionally, *luxI* homologs in the *Arthrobacter* sp. UMCV2 genome were not found. In contrast, 18 predicted protein sequences homologous to *luxR* were localized.

We used a domain-based approach to analyze putative *luxR*-related proteins and compared them with four well-characterized LuxR proteins. We included the first protein described as LuxR, originally found in *A. fischeri* (Fuqua et al., 1994), as a reference protein. PipR from *Pseudomonas* sp. GM79 was included as a reference LuxR since it is a well-experimentally characterized protein belonging to the LuxR protein family that responds to the plant derivative compound *N*-(2-hydroxyethyl)-2-(2-hydroxyethylamino) acetamide through a periplasmic binding protein. PipR has been included in the subfamily PAB that integrates LuxR proteins that respond to low-weight plant molecules (Mosquito et al., 2020; Xu, 2020). The LuxR protein from *Streptomyces purpurogeneiscleroticus* NRRL B-2952 (Rajput and Kumar, 2017) was also included as a reference since it is part of a canonical QS LuxI-LuxR system present in actinobacteria characterized *in silico*. Additionally, RevU was included as a non-canonical LuxR protein from actinobacteria (Panthee et al., 2020) since this protein binds the biomediator BR-1, a β-carboline compound, to regulate the synthesis of reveromycin in *Streptomyces* SN-593. RevU belongs to the LAL-LuxR subfamily (large ATP-binding regulators of the LuxR family), which is characterized by its large size (over 900 a.a. residues) and an ATP-binding domain in the N-terminus. Although it is present in gram-negative bacteria, it is more frequently found in actinobacteria (De Schrijver and De Mot, 1999; Panthee et al., 2020). Canonical LuxR from *A. fischeri* and *S. purpurogeneiscleroticus*, as well as PipR, displayed the same architecture composed of the autoinducer binding domain IPR005143 at the N-terminal end and the DNA-binding domain IPR000792 at the C-terminal. Unsurprisingly, in the LAL-LuxR subfamily member RevU protein, the autoinducer binding domain was replaced by an ATPase domain in addition to the large size of the protein (Figure 3).

None of the *Arthrobacter* sp. UMCV2 sequences showed architectures present in the model luxR sequences, although 11 sequences exhibited the DNA-binding domain IPR000792 (Figure 3). This is a HTH domain that was previously used as a criterion to include proteins in the luxR protein family (De Schrijver and De Mot, 1999; Lopes-Santos et al., 2012; Rajput and Kumar, 2017). We conclude that these 11 proteins belong to the LuxR transcription regulator family and are designated as Auto-inducer-related Regulators (AirR). Among these proteins, eight were grouped in cluster I (Figure 4) and six of them possessed a signal transduction response regulator receiver domain (IPR001789) that substitutes the autoinducer binding domain of canonical LuxR. In other proteins with HTH domains, the autoinducer binding domain is substituted either by an ATPase domain (as happens in RevU) or no amino-terminal domain was identified. Three of these sequences clustered with RevU from *Streptomyces* and LuxR or LuxR related to *Streptomyces iranensis*, *Streptosporangium roseum A. terricola*, and *Arthrobacter* spp., supporting the involvement of these proteins in the LuxR family. A separate group (cluster II of Figure 4) was found in which the canonical DNA-binding HTH domain was substituted with an OmpR/PhoB-type DNA-binding domain together with the same signal transduction response regulator receiver domain present in most of the cluster I AirR sequences (Figures 3, 4). We designated the auto-inducer analog regulator (AiaR) to this group because they form a sister cluster to AirR sequences deep inside the actinobacteria LuxR protein group but do not pose the HTH domain that defines LuxR protein, in addition to the analogous function of both domains with those present in canonical LuxR. Because no Lux-I homologous proteins were found in *Arthrobacter* sp. UMCV2, all AirR constitute “solo” proteins inside the luxR family.

### *Arthrobacter* sp. UMCV2’s compound *N, N*-dimethylhexadecylamine induces auto-inducer-related regulators

LuxR solos have been extensively studied in Gram-negative bacteria. Some LuxR solos bind AHL from other bacterial species, probably as a strategy to expand their regulatory network by utilizing the existing components of the resident quorum-sensing systems (Martínez et al., 2015; Mosquito et al., 2020). Also, other LuxR solos have evolved to respond to different molecules including signals from plants (Patel et al., 2013). Although the functions of these proteins are under-studied in Gram-positive bacteria, especially in actinobacteria other than *Streptomyces*, the phylum has been phylogenetically explored by Lopes-Santos et al. (2012) and revised by Polkade et al. (2016). In *A. aurescens* and *A. chlorophenolicus*, multiple sequences *of luxR* homologs have been reported (16 and 17 sequences, respectively). However, no experimental work has been conducted (Lopes-Santos et al., 2012).

Because AirR proteins do not show the canonical LuxR autoinducer-binding domain, AHL is not expected to be the signal molecule for AirR proteins. However, a previous study revealed that DMHDA induced swarming motility in *Arthrobacter* sp. UMCV2 and simultaneously upregulates the expression of marker genes of the flagellar apparatus (Martínez-Cámara et al., 2020). Since swarming motility is frequently regulated by QS (Krishnan et al., 2012; Nickzad et al., 2015), in this study, swarming motility induction by DMHDA was reproduced and the expression of the *airR* genes was determined. It was found that 10 of the 16 *luxR*-related genes were statistically upregulated (Figure 6), but *airR3* and *airR5* (rich proteins clustered with RevU), and *aiR1*, *airR2*, and *aiR7* were induced over 10-fold. Multiple inductions of *airR* genes suggest the existence of a hierarchical network of *airR* gene induction. In *P. aeruginosa* PAO1, four individual QS circuits have been described. Individual circuits are highly interconnected and involve auto-induction. The induction of an individual circuit by its auto-inductor results in the induction (or repression) of one or more of the other three (Papenfort and Bassler, 2016). Similarly, it is possible that DMHDA induction of an *airR* gene directly or indirectly upregulates other *airR* genes *via* unknown mechanisms. Therefore, additional studies are required to elucidate the function and regulation of *airR* genes.

In this study, the genomic analysis indicated that *Arthrobacter* sp. UMCV2 forms a separate branch within the *genus Arthobacter*, potentially constituting a new species. Genomic analysis also identified a group of 16 genes designed as *airR*, 10 of which were induced by *Arthrobacter* sp. UMCV2 compound DMHDA, which simultaneously induces QS behavior. To the best of our knowledge, this is the first report about the induction of the *luxR* gene family in the *Arthrobacter* genus by a bacterial self-compound.

## Data availability statement

The datasets presented in this study can be found in online repositories. The names of the repository/repositories and accession number(s) can be found below: https://www.ncbi.nlm.nih.gov/genbank/, CP024915.1.

## Author contributions

EV-C and GS: conceptualization. GM-H, EV-C, and JH-S: methodology. GS and GM-H: validation. MC-M, RM-C, and JH-S: formal analysis. MC-M, RM-C, and EV-C: investigation and writing – original draft preparation. EV-C: resources, supervision, project administration. EV-C, GS, and GM-H: writing – review and editing. All authors have read and agreed to the published.

## Footnotes

1
https://www.mg-rast.org

2
https://www.ncbi.nlm.nih.gov/genbank

3
https://tygs.dsmz.de/

4
https://www.patricbrc.org

5
https://www.ebi.ac.uk/interpro/search/sequence
