# Supplementary material for: Comparative genomic and functional analysis of Arthrobacter sp. UMCV2 reveals the presence of luxR-related genes inducible by the biocompound N, N-dimethylhexadecilamine
Source: Front Microbiol. 2022 Oct 31;13:1040932. doi: 10.3389/fmicb.2022.1040932 (PMC9659744; doi:10.3389/fmicb.2022.1040932)
Supplement: Supplementary file 1 [file Data_Sheet_1.docx]

Supplementary Material

| **Table S1. *Arthrobacter* sp. UMCV2 sequencing project information.** | |
| --- | --- |
| Property | **Term** |
| Sequencing platforms | Illumina MiSeq |
| Sequencing libraries | 3 libraries of 1,000 bp, 600 bp and 400-450 bp |
| Number of reads | 3,798,665 sequencing reads |
| Finish quality | High-Quality draft |
| Coverage | 57x |
| Scaffolds | 461 |
| Assembler | Newbler v. 2.9 |
| Gene calling method | NCBI Prokaryotic Genome, Annotation pipeline |
| Bioproject | PRJNA315684 |
| Source Material Identifier | UMCV2 |

| **Table S2. Primers used for qRT-PCR in this work** | | |
| --- | --- | --- |
| **Gene** | **Name** | Sequence |
| airR1 | Aa.airR1F | GCCGTATCCTCACAGAGCAC |
|  | Aa.airR1R | GAGGTCGAAGGTCGTCAGGA |
| airR2 | Aa.airR2F | CCGCCCAGATGAAGCAATGG |
|  | Aa.airR2R | AAGTCCTGGAGGACATCGGT |
| airR3 | Aa.airR3F | CCTACCTCCACACGCTGTC |
|  | Aa.airR3R | ACTGCTGTCGAGTTCATGGG |
| airR4 | Aa.airR4F | GGAGTGCCGAACAAGGAGAT |
|  | Aa.airR4R | TTGCTGCTGTCCGAAGATGA |
| airR5 | Aa.airR5F | TCCCCTTCCTGGTATCACCC |
|  | Aa.airR5R | AAGGACATCACCATGCGGAG |
| airR6 | Aa.airR6F | TACAGCGACAACCTCGGAAG |
|  | Aa.airR6R | CAGTGACTGGACCTCGACCT |
| airR7 | Aa.airR7F | CTGATCCTCGAAGGCGAGC |
|  | Aa.airR7R | GGCATCCGCACATCCATCA |
| airR8 | Aa.airR8F | GGGTGGTGGAAACGGATACC |
|  | Aa.airR8R | ACAACGAGAGAAAGCCTCGG |
| airR9 | Aa.airR9F | GTTCCTCGAAGCACGGAAGA |
|  | Aa.airR9R | GGGCGATGTAGACCTTCTCC |
| airR10 | Aa.airR10F | GTCATCCACCGCTGTGTCA |
|  | Aa.airR10R | GCCCGAGATGGTCTTCGTG |
| airR11 | Aa.airR11F | GAGCTACGACGACGATCAGG |
|  | Aa.airR11R | GCGACGGATCTTCTCGATCA |
| airR12 | Aa.airR12F | AAATCGGGGTCGAGCTGTTC |
|  | Aa.airR12R | GGGTCGGGAAATGTACACGG |
| aiaR1 | Aa.aiaR1F | AGGAACTCCTCAGTGGACTCT |
|  | Aa.aiaR1R | TCGTAGAAACGGTAGCCCTG |
| aiaR2 | Aa.aiaR2F | GTCCTGTCGAAGGCACAGAT |
|  | Aa.aiaR2R | TCCGGGTTCCTGTCGATCTT |
| aiaR3 | Aa.aiaR3F | AGTCGCAGATCCTCGACAAC |
|  | Aa.aiaR3R | GCCCGACGTCGATCTTCTTA |
| aiaR4 | Aa.aiaR4F | TTCGTCGTCAAGCCGTTCAG |
|  | Aa.aiaR4R | AACAGGCTGCGAAACCTCC |

| **Table S3. Number of genes annotated with 25 general COG functional categories.** | | | |
| --- | --- | --- | --- |
| Code | Value | % of total* | Description |
| J | 116 | 3.79 | Translation, ribosomal structure and processing |
| A | 1 | 0.03 | RNA processing and modification |
| K | 161 | 5.26 | Transcription |
| L | 77 | 2.52 | Replication, recombination and repair |
| B | 0 | 0.00 | Chromatin structure and dynamics |
| D | 15 | 0.49 | Cell cycle control, cell division, chromosome partitioning |
| Y | 0 | 0.00 | Nuclear structure |
| V | 27 | 0.88 | Defense mechanisms |
| T | 82 | 2.68 | Signal transduction mechanism |
| M | 81 | 2.65 | Cell wall/membrane/envelope biogenesis |
| N | 18 | 0.59 | Cell motility |
| U | 21 | 0.69 | Intracellular trafficking, secretion and vesicular transport |
| O | 54 | 1.76 | Posttranslational modification, protein turnover, chaperones |
| C | 111 | 3.63 | Energy production and conversion |
| G | 202 | 6.60 | Carbohydrate transport and metabolism |
| E | 208 | 6.80 | Amino acid transport and metabolism |
| F | 62 | 2.03 | Nucleotide transport and metabolism |
| H | 83 | 2.71 | Coenzyme transport and metabolism |
| I | 81 | 2.65 | Lipid transport and metabolism |
| P | 92 | 3.01 | Inorganic transport and metabolism |
| Q | 23 | 0.75 | Inorganic ion transport and metabolism |
| R |  | 7.29 | General function prediction only |
| S | 146 | 4.77 | Function unknown |
| - | 1230 | 38.65 | Not in COGs |

| **Table S4. Genome size of the most related species to *Arthrobacter* sp. UMCV2** | | |
| --- | --- | --- |
| Closely related species | Genome size | Assembly accession ^a^ |
| *Arthrobacter* sp. UMCV2 | 3,435,243 | PRJNA315684 |
| *Arthrobacter sedimenti* MIC A30 ^T^ | 4,422,301 | GCA_011750795 |
| *Arthrobacter ruber* CGMCC 1.9772 ^T^ | 3,650,247 | GCA_002954225 |
| *Arthrobacter cheniae* CGMCC 1.9262 ^T^ | 4,015,930 | GCA_003602275 |
| *Arthrobacter bussei* KR32 ^T^ | 3,619,709 | GCA_009377195 |
| *Arthrobacter agilis* CGMCC 1.15723 | 3,220,798 | GCA_002927255 |
| *Arthrobacter agilis* DSM20550 ^T^ | 3,230,608 | GCA_006494655 |
| *Arthrobacter echini* AM23 ^T^ | 3,169,512 | GCA_004803505 |
| *Arthrobacter frigidicola* CGMCC 1.9882 ^T^ | 4,012,059 | GCA_003602285 |
| *Arthrobacter crusticola* SLN-3T ^T^ | 3,661,073 | GCA_004357995 |
| *Arthrobacter cavernae* PO-11 ^T^ | 4,061,565 | GCA_017368795 |
| *Arthrobacter oryzae* DSM 25586 ^T^ | 4,312,309 | GCA_003634095 |
| *Arthrobacter luteolus* DSM 13067 ^T^ | 3,874,523 | GCA_008973725 |
| ^a^ accession number of GenBank data base. | | |

| **Table S5. List of *Arthrobacter* LuxR-related sequences employed in figure 4.** | | | |
| --- | --- | --- | --- |
| Organism | Name | Accesión number | Reference |
| *Aliivibrio fischeri* | LuxR | P12746 | Devine et al. Biochemistry 27, 837-842 (1988) https://doi.org/10.1021/bi00402a052 |
| *Salmonella enterica* | SdiA* | AAC08299.1 | Smith et al. PLoSOne 3, e2826 (2008) https://doi.org/10.1371/journal.pone.0002826 |
| *Agrobacterium tumefaciens* | TraR | 1L3L_A | Zhang, et al. Nature 417, 971–974 (2002) https://doi.org/10.1038/nature00833 |
| *Agrobacterium vitis* | AvhR* | ACM35646 | Hao et al. J Bacteriol 187, 185-192 (2005) https://doi.org/10.1128/JB.187.1.185-192.2005 |
| *Pseudomonas aeruginosa* | QscR* | 3SZT | Ha et al. Mol Cells 33, 53–59 (2012) https://doi.org/10.1007/s10059-012-2208-2 |
| *Pseudomonas aeruginosa* PAO1 | LasR | NP_250121 | Bottomley et al. J Biol Chem 282, 13592-13600 (2007) https://doi.org/10.1074/jbc.M700556200 |
| *Pseudomonas aeruginosa* PAO1 | RhlR | P54292 | Corral-Lugo et al. Commun Integr Biol 9, 2 (2016) https://doi.org/10.1080/19420889.2016.1156832 |
| *Pseudomonas putida* | PpoR* | AX21483 | Subramoni and Venturi. BMC Microbiol 9, 125 (2009). https://doi.org/10.1186/1471-2180-9-125 |
| *Pseudomonas fuscovaginae* | PfsR | CBI67623 | Mattiuzzo et al. Environ Microbiol. 13, 145-162 (2011) https://doi.org/10.1111/j.1462-2920.2010.02316.x |
| *Pseudomonas* sp. GM790 | PipR | EJN19810 | Coutinho et al. Proc Natl Acad Sci U S A. 115:9785-9790 (2018) https://doi.org/10.1073/pnas.1809611115 |
| *Sinorhizobium meliloti* 1021 | SinR | WP_003534108.1 | Marketon et al. J Bacteriol 184, 5686-5695 (2002) https://doi.org/10.1128/JB.184.20.5686-5695.2002 |
| *Sinorhizobium meliloti* | NesR* | CAC47394 | Patankar and Gonzalez Appl Environ Microbiol 75, 946-955 (2009) https://doi.org/10.1128/AEM.01692-08 |
| *Sinorhizobium meliloti* 8530 | ExpR* | ABC88593 | Glenn et al. J Bacteriol 189, 7077-7088 (2007) https://doi.org/10.1128/JB.00906-07 |
| *Erwinia chrysanthemi* (Dickeya chrysanthemi) | ExpR2* | ABV57378 | Hussain et al. J Bacteriol 190, 1045-1053 (2008) https://doi.org/10.1128/JB.01472-07 |
| *Serratia marcescens* | SmaR | CAB92554 | Thomson et al. Mol Microbiol 36, 539-556 (2000) https://doi.org/10.1046/j.1365-2958.2000.01872.x |
| *Brucella melitensis* | VjbR* | Q8YAY5 | Weeks et al. BMC Microbiol 10, 167 (2010) https://doi.org/10.1186/1471-2180-10-167 |
| *Photorhabdus luminescens* TT01 | PluR* | AGO97061 | Brachmann et al. Nat Chem Biol 9, 573-U573 (2013) https://doi.org/10.1038/nchembio.1295 |
| *Photorhabdus asymbiotica* PB68.1 | PauR* | AJW31137 | Brameyer et al. Proc Natl Acad Sci U S A. 112, 72-577 (2015) https://doi.org/10.1073/pnas.1417685112 |
| *Rhizobium leguminosarum* | BisR* | AAO21111 | Wilkinson et al. J Bacteriol 184, 4510-4519 (2002) https://doi.org/10.1128/JB.184.16.4510-4519.2002 |
| *Stenotrophomonas maltophilia* E77 | SmoR* | AKF42426 | Martinez et al. Front Cell Infect Microbiol. 5, 41 (2015) https://doi.org/10.3389/fcimb.2015.00041 |
| *Rhodopseudomonas palustris* | RpaR | WP_011155889.1 | Schaefer et al. Nature. 454, 595-599 (2008) https://doi.org/10.1038/nature07088 |
| Organism | Name | Accesión number | Reference |
| *Methylobacter tundripaludum* LW13 | MmsR* | QBC27703 | Puri et al. Appl Environ Microbiol 85, e02702-18 (2019) https://doi.org/10.1128/AEM.02702-18 |
| *Arthobacter* sp. UMCV2 | LuxR1 | CVO76_06910 | This work |
| *Arthobacter* sp. UMCV2 | LuxR2 | CVO76_13675 | This work |
| *Arthobacter* sp. UMCV2 | LuxR3 | CVO76_13680 | This work |
| *Arthobacter* sp. UMCV2 | LuxR4 | CVO76_13685 | This work |
| *Arthobacter* sp. UMCV2 | LuxR5 | CVO76_13890 | This work |
| *Arthobacter* sp. UMCV2 | LuxR6 | CVO76_13895 | This work |
| *Arthobacter* sp. UMCV2 | LuxR7 | CVO76_14995 | This work |
| *Arthobacter* sp. UMCV2 | LuxR8 | CVO76_02915 | This work |
| *Arthobacter* sp. UMCV2 | LuxR9 | CVO76_14470 | This work |
| *Arthobacter* sp. UMCV2 | AiaR1 | CVO76_02110 | This work |
| *Arthobacter* sp. UMCV2 | AiaR2 | CVO76_02510 | This work |
| *Arthobacter* sp. UMCV2 | AirR10 | CVO76_09115 | This work |
| *Arthobacter* sp. UMCV2 | AiaR3 | CVO76_10615 | This work |
| *Arthobacter* sp. UMCV2 | AirR11 | CVO76_12145 | This work |
| *Arthobacter* sp. UMCV2 | AiaR4 | CVO76_13490 | This work |
| *Arthobacter* sp. UMCV2 | AirR12 | CVO76_14340 | This work |
| *Streptomyces* sp. SN-593 | RevU* | BAK64651 | Panthee et al. Sci Rep 10, 10230 (2020) https://doi.org/10.1038/s41598-020-66974-y |
| *Streptomyces iranensis* | LuxR | CDR09768.1 | https://www.ncbi.nlm.nih.gov/protein/CDR09768.1 |
| *Streptosporangium roseum* | LuxR | WP_031170323.1 | https://www.ncbi.nlm.nih.gov/protein/WP_031170323.1 |
| *Arthrobacter terricola* | LuxR | WP_165962459.1 | https://www.ncbi.nlm.nih.gov/protein/WP_165962459.1 |
| *Arthrobacter spp* | LuxR related | WP_087071769.1 | https://www.ncbi.nlm.nih.gov/protein/WP_087071769.1 |
| *Arthrobacter* spp | LuxR related | WP_133205185.1 | https://www.ncbi.nlm.nih.gov/protein/WP_133205185.1 |
| Blue sequences have been experimentally characterized, red denotes sequences informatically characterized and black is *Arthrobacter* sp. UMCV2 sequences established in this work. LuxR *solos* are marked with *. | | | |
